# The mucosal immune microenvironment in chronic pharyngitis: epithelial barrier dysfunction, inflammatory persistence, and therapeutic perspectives

**DOI:** 10.3389/fimmu.2026.2000898

**Published:** 2026-10-23

**Authors:** Yao Sun, Zetong Hong, Yiming Guo, Haizhen Cui, Yueyao Jiang, Qian Yu

**Affiliations:** 1 Department of Pharmacy, China-Japan Union Hospital of Jilin University, Changchun, China; 2 School of Pharmaceutical Sciences, Jilin University, Changchun, China

**Keywords:** chronic pharyngitis, epithelial barrier, inflammatory resolution, innate immunity, microbiome, mucosal immunity, neuroimmune signaling

## Abstract

Chronic pharyngitis is a clinically heterogeneous condition in which persistent throat symptoms, visible mucosal abnormalities, and underlying biological processes do not necessarily coincide. This critical narrative review synthesizes evidence from human chronic pharyngitis, experimental injury models, adjacent upper-airway disorders, and other mucosal systems to examine how epithelial barrier function, innate and adaptive immunity, microbial ecology, sensory signaling, and tissue repair may contribute to disease persistence. Experimental studies identify epithelial injury and activation of shared inflammatory and stress-response pathways, while human observations highlight substantial variation in anatomical site, microbial findings, immune organization, and treatment response. Adaptive immune responses appear strongly context dependent; microbial community changes are most informative when linked to organism viability, metabolite production, host sensing, or colonization resistance; and sensory pathways may contribute to symptoms partly independently of measurable inflammation. We propose that persistent disease can arise through three overlapping routes: continued exposure, durable alteration of tissue responsiveness, and incomplete resolution or repair, with microbial and sensory processes acting as modifiers. This framework separates symptom improvement, biological target engagement, and durable restoration of tissue function as distinct therapeutic outcomes. Future studies should prioritize anatomically precise longitudinal cohorts, site-resolved single-cell and spatial profiling, functional perturbation in primary pharyngeal systems, and mechanism-informed trials with follow-up after treatment withdrawal. By linking clinical presentation to local biological function and recovery trajectories, chronic pharyngitis may be reclassified into reproducible biological subgroups that support more precise mechanistic investigation and treatment.

## 1 Introduction

Chronic pharyngitis occupies an evolving position between a clinical syndrome and a biologically defined inflammatory disorder. Patients commonly report persistent throat discomfort, dryness, irritation, foreign-body sensation, or frequent throat clearing, sometimes accompanied by mucosal congestion or other visible abnormalities. These manifestations overlap with several upper aerodigestive conditions, while clinical studies of chronic pharyngitis have used different combinations of symptom duration, examination findings, and exclusion criteria. For example, a recent randomized trial enrolled adults with pharyngeal symptoms or signs persisting for more than three months, whereas an earlier case–control study identified patients with persistent throat pain or irritation after clinical and endoscopic evaluation for alternative causes (1, 2). Chronic pharyngitis therefore encompasses clinically similar presentations that may arise from different biological processes.

Persistent symptoms and persistent mucosal inflammation are related but distinct dimensions of this clinical problem. Studies of patients with persistent throat symptoms demonstrate substantial overlap among throat discomfort, voice disturbance, cough, mucus sensation, and throat clearing (3). The San Diego Consensus likewise distinguishes laryngopharyngeal symptoms from laryngopharyngeal reflux disease and emphasizes objective characterization of reflux when assigning an etiological mechanism (4). From an immunological perspective, this distinction creates an important opportunity to connect clinical phenotype with demonstrable mucosal abnormalities and the biological processes that initiate, amplify, or maintain them.

The pharynx is particularly well suited to a tissue-microenvironment perspective because its mucosal surface is continuously exposed to inhaled and ingested material, resident microorganisms, mechanical forces, and secretions. Barrier integrity and local immunity emerge from interactions among epithelial, stromal, microbial, and immune compartments. Single-cell analysis of human oral mucosa has identified distinct epithelial, stromal, myeloid, and lymphoid populations and linked stromal inflammatory programs to leukocyte recruitment, illustrating how cellular organization shapes mucosal immune responses (5). Anatomical context is equally important within the upper aerodigestive tract. The human tonsil atlas reveals a highly organized secondary lymphoid organ with diverse immune populations (6), while recurrent group A streptococcal tonsillitis has been associated with altered germinal-center T-follicular-helper-cell and antibody responses (7). Together, these studies highlight the biological diversity of neighboring mucosal and lymphoid compartments and the importance of defining the precise anatomical site when interpreting immune findings in chronic pharyngitis.

Chronic pharyngitis also provides a useful clinical setting in which to examine a broader problem in mucosal immunology: similar barrier-associated or inflammatory responses can contribute to host defense, tissue injury, repair, or prolonged tissue adaptation, while symptom burden may follow a partly independent trajectory. We therefore propose a framework that integrates anatomical localization, functional measurements, and longitudinal observation to distinguish these states. Within this framework, persistent disease may arise through different combinations of repeated epithelial injury, sustained immune activation, altered microbial interactions, incomplete tissue recovery, and sensory amplification. These mechanisms are likely to vary across patients and may coexist within the same individual over time.

This review examines how epithelial barrier function, innate and adaptive immunity, microbial ecology, sensory signaling, and tissue repair interact across the spectrum of chronic pharyngitis. We conceptualize persistent mucosal inflammation as a dynamic balance among ongoing exposure, tissue responsiveness, immune regulation, and recovery rather than as a direct consequence of symptom duration. Evidence is considered according to its anatomical and experimental context, with direct human chronic-pharyngitis studies integrated alongside mechanistic models and relevant observations from adjacent mucosal disorders. Therapeutic studies are evaluated in relation to symptom improvement, biological target engagement, and restoration of tissue function. Through this approach, we aim to define testable biological phenotypes that can refine the broad clinical label of chronic pharyngitis and support mechanism-informed investigation and treatment. The integrated framework in **Figure 1** summarizes these proposed interactions and their corresponding experimental questions.

**Figure 1:**
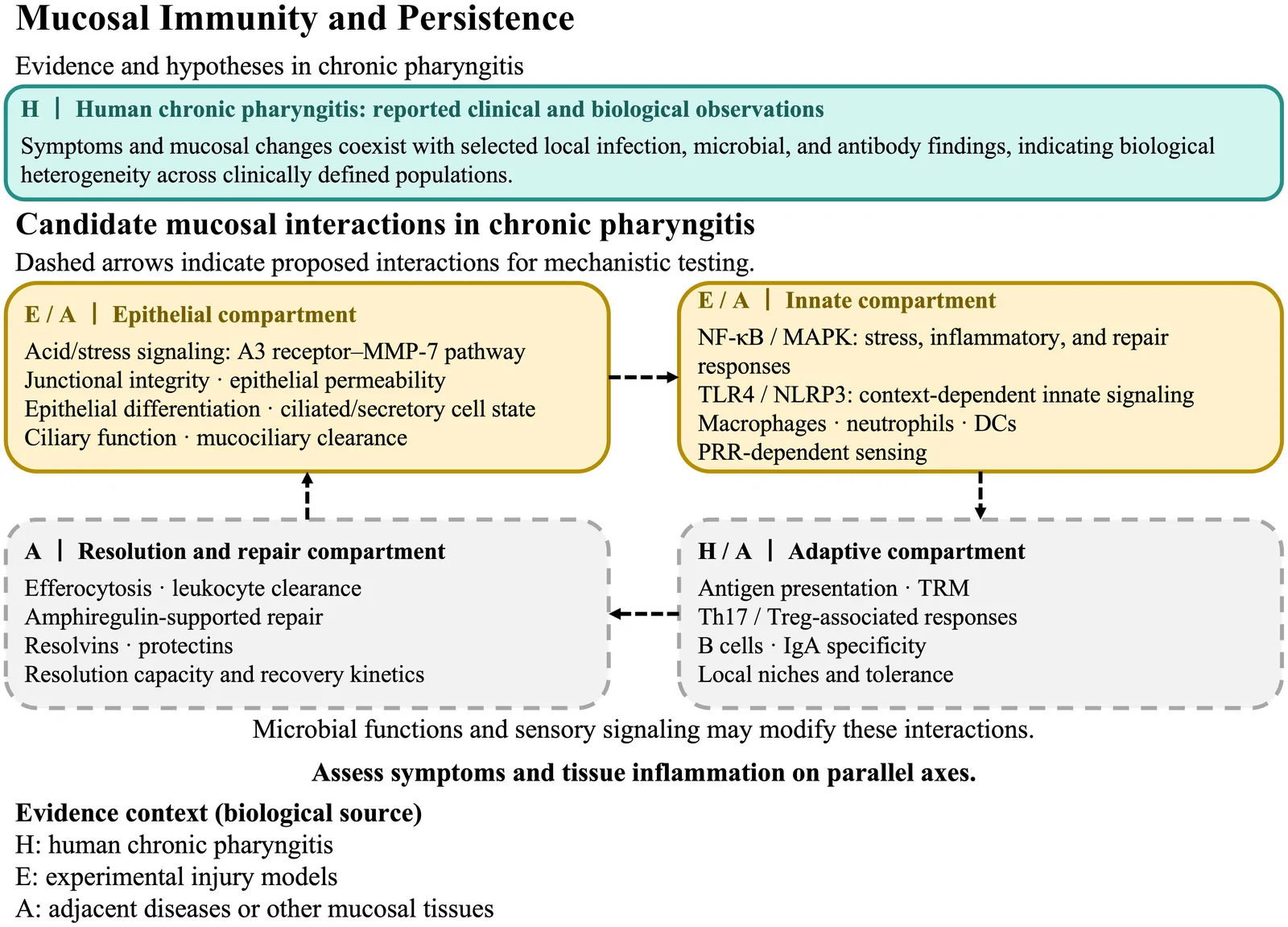
Evidence contexts and proposed mucosal interactions in chronic pharyngitis. H denotes observations reported in human chronic pharyngitis; E denotes experimental injury models; and A denotes evidence from adjacent diseases or other mucosal tissues. These labels indicate biological context rather than evidence quality or causal certainty. Dashed arrows represent proposed interactions among epithelial, innate, adaptive, and resolution/repair compartments that require mechanistic evaluation. Representative mechanisms discussed in Sections 3–8 include epithelial stress signaling, junctional and permeability changes, epithelial differentiation, ciliary function, and mucociliary clearance, innate immune sensing, adaptive immune regulation, and tissue repair pathways (8–13). Microbial functions and sensory signaling are shown as potential modifiers. Symptoms and tissue inflammation are considered parallel outcome dimensions. A3, adenosine A3 receptor; MMP-7, matrix metalloproteinase-7; NF-κB, nuclear factor κB; MAPK, mitogen-activated protein kinase; TLR4, Toll-like receptor 4; NLRP3, NLR family pyrin domain containing 3; DCs, dendritic cells; PRR, pattern-recognition receptor; TRM, tissue-resident memory T cells; Th17, type 17 helper T cells; Treg, regulatory T cells; IgA, immunoglobulin A. Original conceptual schematic; no quantitative data are represented.

## 2 Scope, terminology, and evidence appraisal

### 2.1 Defining the tissue and clinical population

For this review, chronic pharyngitis refers to studies explicitly investigating persistent inflammatory disease of the pharyngeal mucosa or recruiting patients under that diagnosis. Symptom thresholds and examination findings are reported according to individual studies. Recurrent acute pharyngitis, chronic or recurrent tonsillitis, isolated chronic cough, and symptom-defined laryngopharyngeal syndromes are considered related but distinct populations. Chronic epipharyngitis, often described as chronic nasopharyngitis, is considered here as an anatomically specified pharyngeal inflammatory condition rather than assumed to represent a biologically separate disease entity. The nasopharyngeal/epipharyngeal and oropharyngeal compartments differ in epithelial phenotype, exposure, and immune organization, making precise anatomical localization important when interpreting tissue and cellular findings. However, anatomical compartmentalization should not be equated with mutually exclusive disease classification. Clinical studies of chronic epipharyngitis report symptoms that overlap with chronic pharyngeal presentations, including pharyngeal pain or discomfort, cough, sputum, and postnasal symptoms (14, 15). These studies primarily characterize inflammation at the epipharyngeal/nasopharyngeal site and do not establish how frequently inflammatory abnormalities coexist across the nasopharyngeal and oropharyngeal compartments within the same patient. The extent and biological significance of such anatomical overlap therefore remain important questions for future study.

Clinical descriptions such as simple, hypertrophic, or atrophic pharyngitis refer broadly to congested or erythematous mucosa, mucosal thickening with prominent lymphoid follicles, or thin and dry mucosa, respectively. These classifications describe clinical appearance and may represent heterogeneous biological processes, including epithelial stress, microbial exposure, immune activation, altered secretions, or sensory changes. Likewise, tissue obtained during tonsillectomy or laryngeal surgery provides valuable information on regional mucosal biology while reflecting specific clinical indications and anatomical contexts.

We therefore organize the evidence according to four progressively broader contexts: direct human studies of chronic pharyngitis; experimental models specifically addressing chronic pharyngitis; human studies of anatomically adjacent pharyngeal or upper-airway conditions; and mechanistic studies from other mucosal tissues or cellular systems. This sequence reflects clinical and anatomical proximity and is used throughout the review to guide interpretation across experimental settings. **Table 1** summarizes the human evidence, with disease-specific chronic-pharyngitis studies presented before broader upper-airway observations.

**Table 1:** Human studies directly addressing chronic pharyngitis and closely related pharyngeal observations.

Evidence context	Study and population	Design and main observation	Evidence contribution and context
Direct human chronic pharyngitis	12 selected PCR-positive patients (16)	*Chlamydia pneumoniae* was cultured in 4 cases and detected immunohistochemically in posterior pharyngeal tissue in 9 cases.	Provides local tissue and viable-organism evidence for *C. pneumoniae* in a subgroup of patients with chronic pharyngitis.
Direct human chronic pharyngitis	66 tested cases and 62 controls (1)	Case–control serological study identified an association between otherwise unexplained chronic pharyngitis and *C. pneumoniae* IgA seropositivity.	Extends pathogen-specific evidence to a case–control population and complements local microbial observations with systemic antibody measurements.
Direct human chronic pharyngitis	20 patients (17)	Open randomized pilot study reported symptom and cytological improvement after 30 days of high-molecular-weight sodium hyaluronate treatment.	Links a topical intervention with both clinical symptoms and cytological measures of the pharyngeal mucosa.
Human chronic inflammatory pharyngeal disease including chronic-pharyngitis phenotypes	3,465 patients (18)	Fungal involvement was characterized across erythematous–atrophic and hypertrophic chronic-pharyngitis phenotypes; *Candida* species accounted for most diagnosed pharyngomycosis.	Provides large-scale human microbiological evidence across clinically defined chronic-pharyngitis phenotypes.
Direct human chronic pharyngitis	62,823 outpatient records (19)	Time-series analysis identified associations between daily chronic-pharyngitis visits and short-term variation in ambient pollutant exposure.	Provides population-level evidence linking environmental exposure with chronic-pharyngitis presentation.
Direct human chronic pharyngitis	45 patients with exacerbation of chronic catarrhal pharyngitis (20)	Microbiological testing identified several bacterial and fungal taxa before a 14-day course of *Streptococcus salivarius* K12; throat symptoms and halitosis improved during follow-up.	Integrates microbiological characterization with longitudinal clinical assessment in a defined chronic-pharyngitis cohort.
Direct human chronic pharyngitis	22 patients and 8 controls (21)	Exploratory 16S analysis identified differences in selected richness and taxonomic features, while overall beta-diversity separation was not statistically significant.	Provides direct community-level microbial profiling across simple and hypertrophic chronic-pharyngitis phenotypes.
Direct human chronic pharyngitis	Prospective open cohort of patients with chronic pharyngitis (22)	Adjunctive autologous platelet-rich plasma was evaluated alongside symptom trajectories, bacteriological examination, and mass-spectrometric microbial markers obtained from the pharyngeal mucosa before and after treatment.	Provides longitudinal human evidence integrating clinical response with repeated local microbial measurements.
Direct human chronic pharyngitis	103 analyzed patients (2)	Randomized botanical-lozenge trial reported short-term improvement in symptoms and clinical signs compared with starch placebo over 15 days.	Provides controlled therapeutic evidence in a clinically defined chronic-pharyngitis population.
Human upper-airway cohort with a chronic-pharyngitis subgroup	28 patients with inflammatory upper-airway disease, including 11 with chronic pharyngitis (23)	Salivary histatin-associated measurements were compared with low-molecular-weight antimicrobial activity and inflammatory manifestations, with coccoid microbiota assessed in parallel.	Provides human functional evidence linking endogenous salivary antimicrobial activity with inflammatory and microbial measurements in a cohort that included chronic pharyngitis.
Adjacent human pharyngeal evidence	60 patients with Reinke edema (24)	Posterior pharyngeal biopsies identified chronic pharyngitis in 20/40 patients with acid reflux and 5/20 without reflux; the overall difference was not statistically significant.	Provides anatomically relevant pharyngeal tissue observations in a laryngeal surgical population and informs the reflux-associated epithelial-injury context.

Studies are organized according to clinical and anatomical proximity to chronic pharyngitis, with disease-specific human evidence presented before broader upper-airway observations. The table summarizes complementary human evidence spanning pathogen-specific detection, environmental exposure, microbial ecology, endogenous antimicrobial activity, mucosal cytology, and therapeutic investigation. Sample sizes refer to the populations analyzed or described in the original studies. IgA, immunoglobulin A; PCR, polymerase chain reaction; 16S, 16S ribosomal RNA gene.

### 2.2 Evidence appraisal

This review used a critical narrative synthesis approach. A structured update search was conducted in PubMed from database inception through October 7, 2026, without a lower publication-date restriction. The core disease-specific search combined the phrases “chronic pharyngitis,” “chronic hypertrophic pharyngitis,” “chronic atrophic pharyngitis,” “chronic granular pharyngitis,” and “chronic catarrhal pharyngitis” in the title or abstract and retrieved 224 records. A broader sensitivity search using chronic[Title/Abstract] AND pharyngitis[Title/Abstract] was also performed to identify alternative terminology.

The core disease block was combined with prespecified terms covering immune and inflammatory responses, cytokines, lymphoid and myeloid cells, epithelial and barrier biology, microbiota, infection, treatment, randomized trials, and evidence syntheses. The complete search strings and retrieval counts are provided in **Supplementary Table S1**. Reference lists of relevant primary studies and evidence syntheses were also examined to identify additional clinical or mechanistic studies.

Evidence was eligible for synthesis when it addressed chronic pharyngitis directly or contributed mechanistic information relevant to a defined component of the pharyngeal mucosal immune microenvironment. Studies focused on acute pharyngitis were considered selectively when they informed relevant inflammatory mechanisms or provided contextual evidence within reviews encompassing both acute and chronic pharyngitis populations.

Studies were prioritized for detailed discussion when they provided direct tissue, cellular, functional, longitudinal, or intervention-based evidence; clarified the anatomical origin of a biological signal; or contributed a distinct mechanistic or methodological insight. Higher-level evidence, including systematic reviews and meta-analyses, was incorporated where relevant to characterize the broader clinical evidence base. A total of 74 publications were incorporated into this critical narrative synthesis, including primary studies, systematic reviews and other evidence syntheses, and contextual references drawn from the evidence domains described above. **Tables 1**–**3** present selected studies illustrating major evidence categories and translational questions rather than an exhaustive inventory of all cited publications.

**Table 2:** From experimental findings to testable mechanisms.

Evidence context	Reported finding	Mechanistic question and next experiment
Chemical-injury models of chronic pharyngitis (25–27)	Changes in serum mediators and pharyngeal NF-κB, MAPK, PI3K–Akt, or JAK–STAT signaling highlight shared inflammatory, stress-response, survival, and repair networks.	Localize pathway activation to specific cell populations; identify the upstream ligand and receptor; use selective perturbation and rescue to distinguish receptor-dependent sensing from secondary responses to tissue injury; incorporate exposure-withdrawal or rechallenge designs.
Experimental pharyngitis with macrophage assays (28)	Reduced inflammatory mediator production and altered macrophage polarization-associated markers under defined stimulation.	Define the corresponding macrophage programs in pharyngeal tissue and test whether selective macrophage perturbation modifies epithelial injury, microbial control, clearance, or repair.
*Dendrobium officinale* polysaccharides in rat pharyngitis (29)	Pharyngeal TLR4/NF-κB-related inflammatory signaling changed, while occludin and claudin-1 measurements were obtained from the colon.	Measure epithelial integrity and permeability directly in pharyngeal tissue and use compartment-specific measurements to distinguish local from systemic treatment effects.
Primary human pharyngeal epithelial injury model (11)	Acid-associated A3 receptor/MMP-7 signaling and E-cadherin cleavage were attenuated by a plant-extract mixture.	Evaluate pathway activity in clinically characterized pharyngeal tissue and combine target-specific perturbation with permeability, viability, and recovery assays.
Laryngeal reflux tissue and epithelial-cell experiments (12)	Pepsin exposure was associated with ROS/NLRP3/IL-1β signaling, while pharmacological interference with oxidative signaling or NLRP3 attenuated cellular injury.	Localize inflammasome activation in pharyngeal tissue and test its functional contribution using primary cells, selective perturbation, viability controls, and pathway-specific readouts.
Other mucosal injury and repair models (30, 31, 8)	Amphiregulin-mediated tissue protection and pro-resolving pathways illustrate coordination among epithelial repair, leukocyte clearance, and efferocytosis.	Characterize recovery kinetics, efferocytosis, leukocyte clearance, and epithelial repair in longitudinal pharyngeal samples and relate these functions to persistence or recovery.

NF-κB, nuclear factor κB; MAPK, mitogen-activated protein kinase; PI3K, phosphoinositide 3-kinase; Akt, protein kinase B; JAK–STAT, Janus kinase–signal transducer and activator of transcription; TLR4, Toll-like receptor 4; MMP-7, matrix metalloproteinase-7; ROS, reactive oxygen species; NLRP3, NLR family pyrin domain containing 3; IL-1β, interleukin-1β; A3 receptor, adenosine A3 receptor.

**Table 3:** Therapeutic evidence and translational interpretation in chronic pharyngitis.

Intended action and evidence context	What the evidence supports	Next translational step
Reflux-directed therapy: acid suppression — evidence synthesis and persistent-throat-symptom trial (32, 33)	Earlier evidence synthesis suggested symptomatic benefit from proton-pump inhibitors in selected patients with suspected reflux-related laryngopharyngeal symptoms, while also highlighting uncertainty in establishing reflux causation. In the symptom-selected TOPPITS population, lansoprazole did not improve the primary symptom outcome compared with placebo.	Evaluate treatment response in objectively characterized reflux phenotypes using exposure measures, anatomical assessment, and pharyngeal outcomes.
Anatomically targeted local intervention: epipharyngeal abrasive therapy (EAT) — multicenter prospective observational study in chronic nasopharyngitis (34)	In 175 analyzed patients, two months of weekly EAT were accompanied by longitudinal improvement in symptoms and standardized endoscopic inflammatory findings. The prospective multicenter design supports longitudinal assessment in an anatomically defined population, whereas the single-arm design does not establish the causal efficacy of EAT.	Evaluate EAT in controlled studies with anatomically defined eligibility criteria, appropriate comparator groups, local biological endpoints, and follow-up after treatment cessation.
Barrier support: sodium hyaluronate — small direct clinical pilot (17)	Preliminary improvement in symptoms and cytological measures in chronic pharyngitis.	Evaluate the clinical signal in larger controlled trials incorporating functional barrier measurements and follow-up after treatment cessation.
Exposure reduction/barrier support: alginates — epithelial models and reflux-related clinical trials (35–37)	Experimental studies support protection against pepsin–acid-associated epithelial injury; clinical studies in reflux-related populations reported symptom improvement, while placebo-controlled evidence did not demonstrate superiority of alginate over placebo.	Link clinical outcomes to objectively characterized exposure, local epithelial function, formulation properties, and durability of response in defined populations.
Higher-level evidence for anti-inflammatory/botanical formulations — systematic reviews and meta-analyses (38–40)	A chronic-pharyngitis-specific meta-analysis reported clinical response together with pooled IL-2 and TNF-α changes (39). In a mixed-population synthesis, RSI and RFS analyses drew on two chronic-pharyngitis studies, whereas CRP and adverse-event analyses drew on three acute-pharyngitis studies (40). Across the evidence base, methodological quality and safety characterization remain important limitations.	Use standardized chronic-pharyngitis definitions, well-characterized interventions, predefined biological endpoints, systematic safety assessment, and longer-term follow-up in future trials.
Microbial modulation: *Streptococcus salivarius* K12 — direct chronic-pharyngitis, cellular, and adjacent clinical studies (41–43, 20, 44, 45)	A chronic-pharyngitis cohort reported symptom improvement during K12 administration, while experimental and adjacent clinical studies described strain-specific modulation of epithelial responses, pathogen competition, transient salivary enrichment, and variable effects on salivary immune measures.	Test strain-specific clinical effects in defined chronic-pharyngitis phenotypes and connect colonization, microbial function, host response, and clinical outcomes.
Botanical formulation/candidate anti-inflammatory action — direct randomized trial (2)	Short-term improvement in symptom and sign scores with the tested botanical-lozenge formulation in a defined chronic-pharyngitis population.	Replicate the clinical signal with well-matched controls and evaluate local target engagement, formulation-specific effects, recurrence, and longer-term outcomes.
Candidate immune-pathway or pro-resolution interventions — experimental and adjacent-disease evidence (30, 31, 8, 12)	Experimental evidence identifies immune-signaling, tissue-repair, efferocytosis, and resolution pathways that could provide mechanistic targets.	Establish a reproducible local pathway abnormality and functional contribution, identify responsive patient subgroups, and evaluate tissue recovery together with antimicrobial defense.
Sensory/behavioral symptom management — refractory chronic-cough trials (46–48)	Sensory pharmacology and behavioral intervention can modify upper-airway symptom expression in the studied chronic-cough populations.	Evaluate selected approaches in chronic pharyngeal symptom phenotypes while measuring sensory responses, mucosal inflammatory activity, and tissue recovery separately.

Intended action and evidence maturity represent separate dimensions; the categories do not constitute a treatment hierarchy or algorithm. Higher-level evidence syntheses are included alongside individual clinical and experimental studies to contextualize treatment effects, biological outcomes, and recurring methodological limitations. Symptom improvement, biological target engagement, and durable recovery of tissue function are distinct therapeutic outcomes. Precision immunotherapy would require validated local targets and reproducible treatment-responsive endotypes. EAT, epipharyngeal abrasive therapy; TOPPITS, Trial of Proton Pump Inhibitors in Throat Symptoms.

## 3 The epithelial barrier as an organizing interface

### 3.1 Exposure and epithelial injury

An epithelial barrier framework offers a practical starting point because exposure occurs at the mucosal surface, whereas immune cells often respond to the consequences of that exposure. Different levels of evidence, including epidemiological association, tissue injury, and molecular mechanisms, provide complementary information about this process. A time-series analysis of 62,823 outpatient records in Xinxiang linked short-term variation in several ambient pollutants with visits coded as chronic pharyngitis (19). The study supports environmental exposure as a relevant research variable, while individual susceptibility and biological pathways remain to be defined. At the disease-specific experimental level, an ammonia-induced rat model showed pharyngeal mucosal epithelial keratinization or hyperplasia, inflammatory-cell infiltration, and bleeding, with parallel increases in IL-1β, IL-6, and TNF-α; these changes were attenuated by Triphala treatment (49). This model links structural epithelial injury with inflammatory activity in a chronic-pharyngitis model.

Adjacent reflux-related human and experimental studies provide a more developed framework for epithelial injury. In patients with Reinke edema, posterior pharyngeal biopsies were compared between groups with and without acid laryngopharyngeal reflux. Chronic pharyngitis was identified in 20 of 40 patients with reflux and 5 of 20 without reflux; the overall comparison did not reach conventional statistical significance (24). This study illustrates the value of combining objective exposure assessment with anatomical tissue evaluation in selected clinical populations.

At the cellular level, primary human pharyngeal epithelial cells provide a complementary mechanistic system for examining acid-related injury. Increased adenosine A3 receptor and matrix metalloproteinase-7 signaling accompanied E-cadherin cleavage, while a plant-extract mixture attenuated these changes (11). These findings connect acid-related exposure with junctional remodeling in pharyngeal epithelial cells. In rabbits, repeated acid and pepsin exposure produced laryngopharyngeal epithelial changes, reduced E-cadherin expression, and increased intercellular permeability assessed with lanthanum tracing (50). Together, these studies establish experimentally tractable barrier abnormalities and provide a basis for evaluating their relevance across clinically defined chronic pharyngitis populations.

### 3.2 Barrier measurements and immune consequences

E-cadherin is an adherens-junction protein, and its abundance or cleavage captures one component of epithelial barrier integrity. A comprehensive barrier phenotype would combine tissue localization with permeability measurements, epithelial viability, and recovery after injury. The distinction is illustrated by the rabbit reflux study, in which structural abnormalities were detected without a corresponding significant change in transient mucosal impedance (50). Therefore, individual barrier markers should be interpreted according to the biological property they represent.

Barrier function is also dependent on epithelial identity and anatomical site. In respiratory-type epithelium, mucosal defense extends beyond junctional integrity and permeability to include appropriate epithelial differentiation, maintenance of ciliated and secretory cell populations, mucus and airway-surface properties, coordinated ciliary activity, and mucociliary clearance. These functions influence the residence and removal of inhaled particles and microorganisms and thereby shape epithelial exposure and downstream immune signaling (9, 10). Impaired ciliated-cell differentiation or mucociliary transport may therefore represent epithelial dysfunction even when conventional junctional markers are relatively preserved. In site-resolved pharyngeal studies, barrier phenotyping should accordingly be matched to epithelial type: junctional organization and permeability are particularly relevant to stratified squamous mucosa, whereas respiratory-type nasopharyngeal/epipharyngeal epithelium additionally requires assessment of differentiation, ciliary function, mucus properties, and mucociliary transport.

Claudin and occludin findings also require site-specific interpretation: in the Dendrobium rat study, occludin and claudin-1 were measured in the colon, not the pharynx (29). These findings provide information about barrier-associated pathways and highlight the importance of site-specific assessment in human pharyngeal tissue.

Pepsin-related studies further demonstrate how experimental context shapes interpretation. Neutral-pH pepsin exposure damaged organelles and altered stress-related gene expression in FaDu hypopharyngeal carcinoma cells (51). In an immortalized vocal-cord epithelial system, pepsin-associated cell dissociation and E-cadherin cleavage were attenuated by amprenavir (52). These models identify candidate injury mechanisms and pharmacologically modifiable responses for subsequent evaluation in clinically characterized pharyngeal disease.

Epithelial injury may alter access of luminal material to immune sensors and influence mediator release. The temporal relationship between barrier disturbance and immune activation is a key mechanistic question in human chronic pharyngitis. Integrated assessment of permeability, viability, mediator release, and recovery kinetics may help distinguish cytotoxic injury from regulated inflammatory responses and clarify whether epithelial dysfunction contributes to persistent mucosal activation.

## 4 Innate immune signaling and epithelial–myeloid communication

### 4.1 Disease-specific evidence and candidate innate pathways

Animal models labeled as chronic pharyngitis commonly use repeated chemical irritation. These systems reproduce selected features of epithelial injury and inflammation and allow interventions to be compared under controlled exposure. Their mechanistic interpretation depends on the duration and anatomical distribution of the exposure and on whether inflammatory and tissue responses are assessed during exposure or after withdrawal of the irritant. Recovery and withdrawal experiments are therefore particularly informative for distinguishing responses to continuing injury from persistent alterations in the mucosal state.

In an ammonia-induced rat model, Ganluyin reduced inflammatory histological changes, circulating inflammatory mediators, and pharyngeal expression of cyclooxygenase-2 and nuclear factor κB (NF-κB) p65 (25). A complementary study using a group A streptococcus-induced rat chronic-pharyngitis model identified changes in IL-1β, IL-6, TNF-α and Toll-like receptor 4 (TLR4)/myeloid differentiation primary response 88 (MyD88)/NF-κB signaling, providing an additional disease-specific experimental context for innate inflammatory pathways (53). Other studies have combined experimental pharyngitis with macrophage assays. *Citrus grandis* polysaccharides and luteolin, for example, modified inflammatory mediator production and polarization-associated markers under defined *in vitro* stimulation (28). Together, these disease-specific experimental studies identify inflammatory, innate-sensing, and macrophage-related programs that provide a mechanistic foundation for subsequent comparison with adjacent mucosal systems.

Recent work has expanded the candidate signaling network. *Dendrobium officinale* flower polysaccharides altered pharyngeal TLR4/NF-κB-related readouts in a chemically induced rat model (29). *Hosta plantaginea* fractions were associated with changes in Janus kinase–signal transducer and activator of transcription (JAK–STAT), phosphoinositide 3-kinase–Akt (PI3K–Akt), mitogen-activated protein kinase (MAPK), and NF-κB signaling (26). A 2026 study integrating pharyngeal transcriptomics and serum metabolomics likewise implicated MAPK and PI3K–Akt pathways after treatment with a Zhushagen–Shandougen herb pair (27). The convergence of these studies on NF-κB, MAPK, PI3K–Akt, and related pathways highlights a set of shared stress, inflammatory, survival, and repair networks operating in experimental pharyngeal injury.

Human salivary data provide a complementary disease-relevant perspective. In a mixed upper-airway inflammatory cohort that included 11 patients with chronic pharyngitis, salivary histatin-associated measurements were related to clinical inflammation scores and low-molecular-weight antimicrobial activity, while coccoid microbiota were assessed in parallel (23). These observations illustrate the feasibility of integrating endogenous antimicrobial activity, inflammatory phenotype, and microbial measurements in patients with pharyngeal disease.

Across these experimental studies, the cellular source and upstream stimulus of pathway activation are central to mechanistic interpretation. The acid-associated A3 receptor/MMP-7 response described in pharyngeal epithelial cells provides an example of epithelial injury signaling arising outside a canonical pattern-recognition receptor (PRR) pathway (11). Serum cytokines provide information on systemic inflammatory responses, whereas cell-resolved or spatial measurements are needed to localize signaling within pharyngeal tissue. PRR-dependent sensing can be tested by defining the microbial or damage-associated ligand, localizing receptor engagement, and using receptor-specific perturbation or rescue to connect the initiating stimulus with downstream responses.

### 4.2 From pathway association to causal inference

Bulk tissue measurements integrate both changes in cellular state and changes in the abundance of infiltrating or resident cell populations. Similarly, reduced pathway phosphorylation after a multicomponent intervention may reflect decreased tissue injury, altered cellular composition, or direct modulation of signaling. Causal inference is therefore strengthened by cell-resolved localization, selective perturbation, appropriate controls, and rescue experiments linking the proposed target to a defined biological phenotype. **Table 2** pairs commonly reported readouts with experiments that can distinguish among these possibilities.

Pepsin-associated inflammasome research provides a useful adjacent mechanistic example. A study of laryngeal tissues and epithelial cells linked pepsin exposure with reactive oxygen species, NLR family pyrin domain containing 3 (NLRP3)-associated signaling, caspase-1, and interleukin-1β (IL-1β); pharmacological interference with oxidative signaling or NLRP3 attenuated cellular injury (12). These perturbation experiments provide a mechanistic precedent for connecting an epithelial exposure to inflammasome-associated injury. In chronic pharyngitis, the corresponding hypothesis can be evaluated through local assessment of inflammasome activation, cellular localization, and functional perturbation in primary pharyngeal systems.

Macrophage biology requires similar attention to cellular state and function. Macrophage activation is better understood in terms of stimulus- and context-dependent programs than as fixed, mutually exclusive “M1” and “M2” identities (54). In chronically exposed mucosa, antimicrobial activity, inflammatory mediator production, cellular clearance, and repair functions may coexist or change over time. The mechanistically informative questions are therefore which macrophage programs are present, where they are localized, how they evolve during disease and recovery, and how they influence epithelial function.

Human oral mucosal single-cell data further broaden this cellular framework by identifying epithelial and stromal programs associated with neutrophil recruitment and tissue immune organization in periodontitis (5). These findings support consideration of fibroblasts, endothelial cells, and other stromal components alongside epithelial and myeloid populations when investigating the pharyngeal immune microenvironment. Integrating spatial and single-cell measurements with functional experiments could therefore reveal how epithelial, stromal, and myeloid compartments coordinate persistent inflammation and recovery.

## 5 Adaptive immunity and local immune organization

### 5.1 Context-dependent T-cell responses

Adaptive immunity may contribute to sustained pharyngeal inflammation through antigen-specific responses, altered immune regulation, or repeated recruitment to injured tissue. A useful framework for investigating adaptive immunity in chronic pharyngitis integrates T-cell phenotype, antigen specificity, and tissue localization. Th17- and regulatory T-cell (Treg)-associated responses are particularly informative because their biological effects depend strongly on anatomical context, antigen exposure, and tissue state.

Adjacent mucosal studies illustrate this context dependence. In the gingiva, physiological mechanical damage associated with mastication induced epithelial interleukin-6 (IL-6) and supported local Th17 accumulation independently of commensal colonization (55). In a murine intranasal group A *Streptococcus* model, IL-6-dependent Th17 responses contributed to protective immunity and bacterial clearance (56). Human genetic studies of IL-17RA and IL-17F deficiencies further establish an important role for IL-17 signaling in protection against mucocutaneous *Candida* infection (57). Together, these observations show that Th17-associated immunity can participate in barrier defense as well as inflammatory tissue responses, making cellular source, antigenic context, and functional outcome essential to interpretation in chronic pharyngitis.

Tissue-resident memory T cells (TRM) provide a complementary framework. In mice, nasal CD8-positive TRM limited influenza spread to the lung (58). More recent work identified Th17-enriched nasal CD4-positive TRM in human and mouse tissues, with protective function evaluated in murine influenza challenge (13). These studies highlight the potential for persistent local T-cell populations to support rapid mucosal protection. In chronic pharyngitis, resident phenotypes and T-cell receptor clonotypes could be linked to anatomical location, persistence, antigen responsiveness, and effects on epithelial function. Combining CD69 or CD103 expression with spatial localization, transcriptional phenotype, and clonotype tracking would provide a more complete assessment of tissue residence and function. Local antigen presentation is another informative variable: dendritic-cell or epithelial human leukocyte antigen (HLA) class II expression can be integrated with costimulatory profiles and functional T-cell assays to assess antigen-specific activation.

An adjacent clinical study of chronic epipharyngitis reported increased CD4-positive cells in epipharyngeal abrasive samples and a reduction after repeated epipharyngeal abrasive therapy (EAT), alongside symptom improvement (59). The study illustrates the feasibility of local cellular sampling and longitudinal assessment during treatment. More recent multicenter prospective evidence extends this clinical context. Kumai et al. enrolled 219 patients with chronic nasopharyngitis, of whom 175 were included in the final analysis, and prospectively assessed symptoms and standardized endoscopic inflammatory findings during weekly EAT over two months (34). Both symptom scores and endoscopic inflammatory findings improved during follow-up. Because the study was single-arm and observational, without an untreated or sham-treated comparator, these changes should be interpreted as longitudinal associations rather than causal evidence of therapeutic efficacy. The study nevertheless demonstrates the feasibility of linking an anatomically defined nasopharyngeal inflammatory phenotype with standardized local assessment and repeated intervention.

A recent spatial transcriptomic study provides a complementary site-resolved human example. Using Visium HD, Nishi et al. analyzed epipharyngeal tissue from three patients with long COVID and two non-COVID controls and resolved spatially organized epithelial and nonepithelial cell populations together with localized immune and signaling programs (60). Because the study was conducted in the specific context of long COVID and involved a small number of participants, its disease-associated molecular findings should not be generalized to chronic pharyngitis. Its relevance here is anatomical and methodological: it demonstrates that epithelial and immune compartments can be resolved directly within human epipharyngeal tissue and provides a precedent for comparable spatial analyses across chronic pharyngeal inflammatory phenotypes.

Nasopharyngeal mucosa can include adenoid-associated lymphoepithelium with specialized lymphocyte–epithelial interactions (61). Age, organized lymphoid tissue, sampling depth, and anatomical site are therefore important determinants of the cellular populations recovered from upper-airway mucosa.

### 5.2 Tonsillar and humoral immunity in regional context

Tonsillar studies provide powerful examples of adaptive immune mechanisms operating within the upper airway. Children with recurrent group A streptococcal tonsillitis showed abnormalities in germinal-center T-follicular-helper cells, lower antibody responses to streptococcal pyrogenic exotoxin A, and associations with HLA class II variation (7). The integration of cellular phenotype, antibody responses, and host genetics illustrates how anatomically organized lymphoid tissue can shape pathogen-specific immune responsiveness. These findings provide a regional mechanistic reference for investigating adaptive immune organization in chronic pharyngitis.

B-cell and plasma-cell niches connect antigen recognition with local antibody production, while class switching, antibody secretion, epithelial transport, and antigen specificity represent distinct components of humoral immunity. In human intestinal systems, bacterial Toll-like receptor stimulation induced epithelial secretion of a proliferation-inducing ligand (APRIL) and supported T-cell-independent immunoglobulin A2 (IgA2) class switching (62). This mechanism provides a model for examining epithelial–B-cell communication at other mucosal surfaces. In pharyngeal studies, local class-switch activity, plasma-cell abundance, antibody specificity, and epithelial transport can therefore be evaluated as separate but related processes. Antibody detected at the pharyngeal surface may derive from salivary glands, local plasma cells, or neighboring lymphoid tissue, making anatomical localization particularly informative.

Secretory immunoglobulin A (sIgA) is an attractive candidate marker of mucosal immune function. In physically active young adults, a small randomized study reported changes in salivary immune measures after *Streptococcus salivarius* K12 supplementation, without a between-group difference in upper respiratory infection episodes (45). A separate placebo-controlled study found no increase in salivary sIgA levels or salivary flow relative to placebo after K12 administration (44). These studies illustrate how sampling conditions, secretion rate, population characteristics, and clinical outcomes influence the interpretation of salivary immune measurements.

Future studies can strengthen humoral and cellular phenotyping by linking immune identity to function. For T cells, relevant measures include antigen responsiveness, cytokine production after standardized stimulation, clonotype structure, and proximity to damaged epithelium. For B cells and antibodies, antigen specificity and neutralizing or antimicrobial activity may be more informative than total concentration alone. The human tonsil atlas provides a valuable reference for cell annotation (6), while site-resolved pharyngeal profiling could define which adaptive immune states occur at the symptomatic mucosal surface and how they relate to organized lymphoid tissue.

### 5.3 Lymphoid organization and immune tolerance

Tertiary lymphoid structures (TLS) are inducible lymphoid aggregates that arise outside constitutive secondary lymphoid organs. In neonatal mouse lung inflammation, IL-17-dependent CXCL13 induction supported the formation of inducible bronchus-associated lymphoid tissue (63). This provides a mechanistic framework for distinguishing inducible lymphoid organization from constitutive tonsillar or adenoidal tissue. In pharyngeal studies, ectopic lymphoid organization can be assessed through spatially resolved B- and T-cell territories, follicular stromal networks, chemokine expression, and evidence of local immune activity. The relationship between organized lymphoid structures and diffuse pharyngeal mucosal inflammation may therefore represent an important axis of local immune organization.

Antigen-specific immunity and immune tolerance provide a second adaptive dimension. An older study of 29 patients with chronic hypertrophic pharyngitis and 18 healthy controls reported pharynx-specific antigen, corresponding antibodies, and immune complexes in serum or oral fluid (64). These observations suggest that tissue-antigen exposure and local or systemic antibody responses may accompany chronic mucosal inflammation. Future studies could define the relevant antigens, determine the anatomical source of antibody production, and examine whether antigen-specific responses influence epithelial injury or recovery. Treg phenotypes can similarly be paired with suppressive capacity, cytokine production, and tissue-repair functions to distinguish numerical changes from functional immune regulation.

## 6 Microbial ecology, colonization, and infection

The pharyngeal microbiota may influence local immune tone through direct host–microbial interactions, metabolite production, and modification of the epithelial environment. At the same time, secretory conditions, inflammation, treatment exposure, oral health, and diet can reshape microbial communities. Microbial composition and mucosal state are therefore best considered as interacting components, with functional measurements helping to distinguish microbial drivers from ecological responses to disease.

Direct human chronic-pharyngitis studies provide several complementary views of the local microbial environment. A small 16S study compared 11 patients with chronic simple pharyngitis, 11 with chronic hypertrophic pharyngitis, and eight healthy controls. Several richness or taxonomic comparisons differed, whereas overall beta-diversity analyses showed no significant separation among groups (21). A much larger clinical series characterized fungal involvement across erythematous–atrophic and hypertrophic chronic-pharyngitis phenotypes, with *Candida* species accounting for most diagnosed pharyngomycosis (18). In 45 patients with exacerbation of chronic catarrhal pharyngitis, microbiological testing identified several bacterial and fungal taxa before a 14-day course of *Streptococcus salivarius* K12, while throat symptoms and halitosis were followed longitudinally (20). A more recent prospective open cohort incorporated bacteriological examination and mass-spectrometric microbial markers from the pharyngeal mucosa into longitudinal assessment of patients receiving platelet-rich plasma as adjunctive therapy (22). Together, these studies show that microbial composition and local microbial measurements can be integrated directly with clinical phenotype and longitudinal assessment in chronic pharyngitis.

A complementary distinction is between microbial community ecology and pathogen-specific infection. In otherwise unexplained chronic pharyngitis, a case–control study reported an association with *Chlamydia pneumoniae* IgA seropositivity (1). An earlier series enriched for PCR-positive patients provided complementary tissue-level evidence: *C. pneumoniae* was cultured in four of 12 cases and detected immunohistochemically in posterior pharyngeal tissue in nine (16). These studies illustrate an evidence spectrum from systemic antibody responses to local organism detection and viable recovery. Pathogen-specific investigation can therefore integrate clinical phenotype with local sampling, organism viability, tissue localization, and host response rather than relying on symptoms or community composition alone.

Adjacent upper-airway studies add physiological context to these disease-specific observations. In a study of 36 patients with laryngopharyngeal reflux and 39 controls, salivary microbial differences occurred alongside changes in salivary flow and selected protective factors, while sIgA concentrations did not differ significantly between groups (65). Such observations highlight the value of profiling microbial communities together with the local secretory, physicochemical, and immune environment.

For microbial functions that have not yet been characterized directly in chronic pharyngitis, broader mucosal systems provide experimentally tractable mechanisms that can be tested at the pharyngeal surface. Microbial metabolites provide one such link between community structure and host response. In intestinal systems, butyrate supported hypoxia-inducible factor-dependent epithelial barrier function (66), whereas gingival experiments found that butyrate disrupted epithelial junctions and induced inflammatory cell death (67). These contrasting effects illustrate the strong dependence of metabolite activity on tissue context, concentration, and cellular state. *Lactobacillus*-derived indole-3-aldehyde activated the aryl hydrocarbon receptor/IL-22 axis and supported resistance to *Candida* in mouse mucosal models (68). In pharyngeal studies, integrating metabolite profiling with source-resolved microbial analysis and exposure-relevant epithelial assays could connect local metabolite production with barrier integrity, antimicrobial responses, and cell viability.

Microbial sensing provides a second functional layer. Toll-like receptor (TLR)- and nucleotide-binding oligomerization domain (NOD)-dependent pathways can be investigated by linking defined microbial ligands to receptor engagement and downstream cellular responses. Taxonomic profiling, ligand measurements, receptor localization, and perturbation experiments can therefore be combined to distinguish microbial sensing from broader stress-associated NF-κB activation. Colonization resistance can similarly be evaluated by testing whether resident strains or their products restrict pathogen growth, adherence, or epithelial access.

Biofilm-associated persistence represents a spatial form of microbial organization. Microscopy of surgically removed diseased tonsils has demonstrated biofilm-like bacterial communities (69). As an anatomically adjacent lymphoid tissue, the tonsil provides a regional precedent for investigating structured microbial communities at symptomatic pharyngeal sites. Local spatial imaging, viable microbial recovery, and paired host-response measurements could determine whether persistent surface communities are associated with epithelial injury or immune activation in chronic pharyngitis.

Functional microbiology may be particularly useful for translating microbial observations into mechanism. *Streptococcus salivarius* K12 modified epithelial inflammatory responses in a bronchial epithelial system (41), while K12 and M18 interfered with pneumococcal growth or adherence through partly distinct mechanisms in experiments involving Detroit 562 pharyngeal carcinoma cells (42). These mechanistic studies complement the clinical K12 observations described above and illustrate strain-specific pathways through which commensal organisms may influence epithelial responses or pathogen competition. A pediatric randomized study found transient salivary enrichment after K12 administration with little change in overall microbial diversity (43). Future studies could connect microbial abundance with absolute load, viability, functional activity, host responses, and persistence after treatment while accounting for dental status, recent antibiotics, diet, and sampling site.

## 7 Sensory signaling and the interpretation of symptoms

Sensory pathways are relevant because pharyngeal discomfort, throat clearing, and related symptoms can persist across different degrees of measurable mucosal inflammation. Within an immunological framework, sensory signaling can be considered as a parallel biological dimension that both responds to tissue injury and, in some settings, modulates local immune activity.

In experimental bacterial pneumonia, transient receptor potential vanilloid 1 (TRPV1)-positive nociceptors influenced neutrophil and γδ T-cell responses, with calcitonin gene-related peptide contributing to suppression of protective immunity (70). This work provides a mechanistic example of bidirectional communication between sensory neurons and immune defenses in the respiratory tract. Comparable interactions in the pharynx could be examined through spatial localization of nerve–immune contacts, neuropeptide profiling, and functional assays that distinguish sensory excitability from immune-cell activation.

Clinical cough studies illustrate a complementary sensory mechanism. Phase 3 trials of the P2X3 antagonist gefapixant reported reductions in cough frequency in refractory or unexplained chronic cough, with taste disturbances among the most frequently reported adverse events (48). These findings demonstrate that modulation of sensory signaling can alter symptom expression in appropriately selected patients. In chronic pharyngitis, sensory outcomes would be most informative when interpreted alongside measurements of mucosal inflammation and tissue state.

A useful research design would therefore measure symptoms and tissue biology on parallel axes. Symptom improvement with little change in local inflammatory measures may indicate an important sensory component, whereas biological improvement accompanied by persistent symptoms may reflect ongoing sensitization or another symptom-generating pathway. Tracking these dimensions separately can clarify treatment effects and is particularly important in trials that rely heavily on discomfort or throat-clearing scores.

## 8 Why inflammation might persist after the initiating exposure

### 8.1 Continued injury, altered tissue state, and incomplete repair

Persistent mucosal disease may arise from continued exposure, incomplete recovery between exposures, or a tissue state that remains altered after the original stimulus has diminished. These mechanisms make distinct temporal and experimental predictions. Continued exposure predicts improvement when the relevant exposure is reliably reduced, whereas a durable alteration in tissue state predicts persistent functional abnormalities in ex vivo systems after removal from the original environment. Longitudinal sampling, exposure withdrawal, and experimental rechallenge in model systems can therefore help distinguish among these trajectories.

Human chronic rhinosinusitis provides an example of persistent epithelial adaptation. Respiratory basal progenitor cells retained features of prior IL-4/IL-13 exposure in culture, supporting a form of epithelial inflammatory memory (71). This work provides a mechanistic precedent for epithelial states shaped by prior inflammatory exposure that persist beyond the immediate inflammatory environment. In chronic pharyngitis, an analogous phenomenon could be investigated using primary pharyngeal cultures with standardized passage, controlled withdrawal and re-exposure, and assessment of differentiation, barrier function, and inflammatory responsiveness.

A complementary mechanism is repeated injury with incomplete recovery between exposures. Chemical-irritation models are well suited to examining this process when tissue responses are followed during exposure, recovery, and rechallenge. Withdrawal experiments can define recovery kinetics, whereas rechallenge can reveal altered sensitivity to subsequent injury. These temporal phenotypes may distinguish prolonged healing, recurrent injury, and durable tissue adaptation more effectively than a single designation of “chronic” inflammation.

### 8.2 Resolution as an active biological process

Resolution is an active biological program involving removal of dying cells, regulation of leukocyte recruitment, restoration of epithelial organization, and recovery of tissue function. Experiments in influenza-injured lung showed that innate lymphoid cells supported epithelial integrity through amphiregulin (30). Separate work demonstrated a tissue-protective function of Treg-derived amphiregulin that could be dissociated from conventional suppression of immune responses (31). Together, these studies illustrate how immune cells can participate directly in tissue repair as well as inflammatory regulation.

In a murine bacterial pneumonia model, aspirin-triggered resolvin D1 enhanced bacterial clearance and macrophage efferocytosis and accelerated neutrophil clearance (8). A separate influenza study identified reduced protectin D1 production during severe infection and demonstrated antiviral and tissue-protective effects in experimental systems (72). These lipid-mediator studies illustrate how microbial clearance, leukocyte removal, and tissue protection can be coordinated within the same recovery program.

In chronic pharyngitis, resolution biology provides a testable framework for asking whether persistent disease reflects incomplete clearance of inflammatory cells or debris, delayed epithelial recovery, or altered mediator kinetics after the initiating exposure has diminished or been removed. Longitudinal measurement of efferocytosis, lipid mediators, epithelial repair, and tissue function could therefore distinguish persistent inflammatory activity from delayed or incomplete recovery.

The proposed framework thus includes three potentially overlapping routes to persistence: continued exposure, durable alteration of tissue responsiveness, and incomplete resolution or repair. Temporal sampling before, during, and after intervention can help determine which trajectory predominates in an individual or experimental system. Recurrence after treatment withdrawal, recovery kinetics, and responses to experimental rechallenge in model systems may be especially informative for distinguishing transient suppression of inflammatory readouts from restoration of a stable mucosal state.

## 9 Therapeutic perspectives and the strength of translational evidence

### 9.1 Matching therapeutic interpretation to population and mechanism

The central therapeutic challenge is to connect clinical benefit with the population and biological process actually studied. Evidence from an experimental exposure model, a symptom-selected throat cohort, and patients with documented pharyngeal inflammation addresses different therapeutic questions. **Table 3** groups interventions according to their intended action: exposure reduction or barrier support, microbial modulation, candidate immune-pathway modification, and sensory or behavioral symptom management. Evidence maturity provides a separate dimension, ranging from mechanistic plausibility to clinical efficacy and biologically validated treatment response. Precision immunotherapy would require reproducible endotypes linked to treatment-responsive pathways.

Therapeutic outcomes can likewise be considered at several levels. Symptom improvement reflects clinical benefit, immune-state modification reflects measurable change in local immune activity, and homeostatic recovery additionally involves restoration of tissue function and durable mucosal resilience. Assessing these dimensions separately can clarify whether an intervention primarily modifies symptoms, biological activity, or mucosal resilience.

The TOPPITS trial randomized 346 adults with persistent throat symptoms to lansoprazole or placebo and found no significant benefit for the primary symptom outcome (33). This study did not support a symptomatic benefit of empirical acid suppression in an unselected symptom-defined population, reinforcing the importance of identifying the relevant exposure phenotype before treatment. Similarly, clinical improvement after an empirical intervention is most informative when accompanied by evidence that the proposed exposure or biological target has changed. An earlier review of reflux-related chronic pharyngitis and laryngitis provides complementary context: although meta-analytic evidence suggested symptomatic benefit from proton-pump inhibitors in selected patients with suspected reflux-related laryngopharyngeal symptoms, the review also emphasized the difficulty of establishing gastroesophageal reflux as the cause of chronic pharyngeal or laryngeal symptoms using available diagnostic approaches (32).

Anatomically targeted local treatment provides a complementary example of population- and mechanism-specific interpretation. In a multicenter prospective observational study of chronic nasopharyngitis, 175 of 219 enrolled patients were included in the final analysis after two months of weekly EAT, with longitudinal improvement reported in both symptom scores and standardized endoscopic inflammatory findings (34). The prospective multicenter design and paired clinical–endoscopic assessment strengthen the characterization of the treated population and response trajectory. However, the absence of a concurrent control group prevents attribution of the observed changes specifically to EAT. Controlled studies incorporating anatomically matched eligibility criteria, local biological outcomes, and follow-up after treatment withdrawal would provide a stronger test of treatment-specific effects.

For future chronic pharyngitis trials, exposure management should be incorporated into the study design rather than treated as background advice. If an intervention combines a topical product with changes in irritant exposure, implementation and adherence should be measured separately. Patients with a defined infection, reflux phenotype, or other identifiable disorder can be analyzed according to that biological context. This approach allows therapeutic response to be interpreted within mechanistically meaningful subgroups without assuming a single inflammatory pathway across all patients with chronic throat symptoms.

### 9.2 Barrier support and topical approaches

Barrier-supportive approaches provide a direct way to target the symptomatic mucosal surface. An open randomized pilot study involving 20 patients reported symptom and cytological improvement after 30 days of high-molecular-weight sodium hyaluronate (17). The study provides a preliminary clinical and cytological signal that can be evaluated further in larger controlled trials incorporating functional measurements of epithelial integrity and durability of response.

Alginate studies illustrate how mechanistic and clinical evidence can complement one another. In cultured esophageal and vocal-cord epithelial systems, alginate preparations protected against pepsin–acid-associated permeability changes (37). In a randomized study of 50 patients selected using reflux symptom and finding scores, both magnesium alginate and omeprazole groups improved (36). An 80-patient placebo-controlled trial likewise reported improvement in both the alginate and placebo groups, without a significant between-group difference in the primary symptom outcome (35). Together, these studies support continued evaluation of topical barrier and exposure-limiting strategies while highlighting the value of pairing symptom outcomes with objective exposure and epithelial-function measurements.

Local delivery is attractive because it can expose the symptomatic mucosa directly, but formulation is itself part of the intervention. Residence time, swallowing, salivary dilution, tolerability, and the physical properties of a lozenge or viscous vehicle can influence tissue exposure and symptom response. Matched vehicle controls and pharmacologically informed formulation studies are therefore particularly useful for separating delivery effects from target-specific activity. Follow-up after treatment cessation can further determine whether epithelial or symptomatic benefits persist beyond direct product contact.

### 9.3 Anti-inflammatory formulations and immune-pathway modification

Higher-level evidence provides useful context for interpreting the chronic-pharyngitis intervention literature. A systematic review and meta-analysis of 13 randomized trials involving 1,642 patients with chronic pharyngitis reported higher overall clinical response with Lanqin oral liquid together with pooled increases in IL-2 and reductions in TNF-α. The authors also identified generally poor methodological quality among the included trials, and safety was not evaluated in the included randomized studies (39). A more recent systematic review and meta-analysis evaluating Lanqin oral liquid combined with Western medicine included 12 studies, comprising eight chronic-pharyngitis and four acute-pharyngitis studies. The chronic-pharyngitis analyses of the Reflux Symptom Index and Reflux Finding Score were based on two studies, whereas the pooled analyses of C-reactive protein and adverse events each drew on three acute-pharyngitis studies (40). These findings provide complementary clinical evidence across pharyngitis populations, but the inflammatory and safety outcomes from acute-pharyngitis studies should not be interpreted as chronic-pharyngitis-specific evidence. Broader Cochrane evidence on Chinese herbal medicines for sore throat included limited chronic-pharyngitis evidence within a wider pharyngitis evidence base and likewise identified substantial limitations in trial quality (38). Together, these syntheses show that clinical outcomes and selected inflammatory measures have been evaluated in chronic-pharyngitis studies, while evidence from acute-pharyngitis populations provides complementary but distinct clinical context. They also highlight priorities for future trials, including rigorous disease definition, well-characterized interventions, predefined biological endpoints, systematic safety assessment, and evaluation of the durability of response.

A randomized trial of botanical lozenges analyzed 103 patients with chronic pharyngitis and reported improved symptom and sign scores after 15 days compared with a starch placebo (2). This trial provides an additional short-term clinical signal in a defined chronic-pharyngitis population and complements the broader intervention evidence summarized above. Future studies could connect this response with local target engagement, epithelial or immune readouts, recurrence after treatment cessation, and comparative effectiveness. Differences in taste and adherence reported in the full text are also relevant when designing future blinded trials.

Preclinical multi-component formulations provide additional mechanistic leads. Yanshu reduced experimental inflammation and showed *in vitro* antibacterial activity. In separate mouse assays, it did not significantly alter thymus or spleen indices, leukocyte or lymphocyte counts, or the monocyte–macrophage phagocytic index compared with vehicle controls (73). Chemical standardization, exposure characterization, and component-level perturbation could help identify the components and mechanisms responsible for the observed anti-inflammatory and antibacterial effects.

Candidate immune-pathway modification represents a distinct translational stage from phenotype-selected precision immunotherapy. NF-κB, MAPK, JAK–STAT, and inflammasome-associated changes identified in experimental models provide potential mechanistic targets, but these pathways also participate in host defense, epithelial survival, and tissue recovery. Human IL-17 deficiency syndromes, for example, demonstrate the importance of IL-17 signaling in mucosal antimicrobial protection (57). A translational development sequence would therefore connect a reproducible local pathway abnormality with functional contribution, identify the patient subgroup in which it is active, and evaluate both tissue recovery and infection-related consequences during pathway modification.

### 9.4 Microbial and sensory interventions

Microbial interventions are most informative when evaluated at the strain and function level. Clinical studies can define the enrolled phenotype, quantify exposure and persistence of the administered organism, and determine whether changes in microbial function or host response mediate clinical benefit. This framework moves beyond generic “microbiome restoration” toward specific mechanisms such as colonization resistance, pathogen competition, metabolite production, or modulation of epithelial signaling.

Sensory approaches may be particularly relevant when symptom burden and mucosal inflammatory activity are only partly coupled. Randomized trials have reported benefit from gabapentin and speech-pathology interventions in refractory chronic cough (46, 47). These studies provide proof of principle that upper-airway symptom expression can be modified through neural and behavioral pathways. In chronic pharyngitis, such approaches could be evaluated alongside mucosal inflammatory and epithelial measures so that symptom relief, sensory responsiveness, immune activity, and tissue recovery are assessed as distinct but complementary outcomes.

## 10 Discussion and research priorities

### 10.1 A unifying framework with falsifiable alternatives

The available evidence supports a framework in which persistent pharyngeal disease emerges from interactions among exposure, tissue response, and recovery, with the relative contribution of each varying across patients. Epithelial injury and inflammatory signaling are repeatedly observed in experimental systems relevant to the pharynx, while direct human studies demonstrate the feasibility of tissue assessment, microbial sampling, and controlled therapeutic investigation in chronic pharyngitis. Adaptive immune organization, microbial function, sensory signaling, and resolution biology provide additional candidate dimensions that can be tested within this framework rather than assumed to define a single shared endotype.

This framework has practical advantages over a catalog of elevated cytokines. It distinguishes initiating events from maintaining mechanisms and makes divergent findings biologically informative. Normal barrier function despite persistent symptoms would weaken a barrier-centered explanation for that subgroup. Persistent barrier impairment after verified exposure reduction would support investigation of altered repair or tissue state. A microbial association that diminishes after accounting for salivary flow or oral disease would suggest that local environmental factors partly explain the observed association. Specifying such alternatives before data collection can turn descriptive profiling into hypothesis-driven investigation.

The field also needs to distinguish severity markers from actionable mechanisms. A mediator may correlate with tissue damage because it is released by damaged cells, recruited leukocytes, or a compensatory repair response. Treatment-related normalization may strengthen its value as a response marker, whereas causal perturbation is needed to establish whether modifying that mediator changes disease biology. Temporal sampling, cell-resolved localization, and perturbation experiments therefore provide complementary routes from association to mechanism, even when molecular signals are reproducible across multiple platforms.

### 10.2 Build anatomically precise, longitudinal cohorts

The first priority is a reproducible clinical and sampling framework. Studies should report symptom duration and trajectory, the exact site and appearance of mucosal abnormalities, relevant exposures, recent infections, oral and dental status, medication use, and overlapping nasal, laryngeal, or esophageal conditions. Reflux should be characterized according to the biological question being tested rather than inferred solely from symptom scores. Participants with persistent symptoms but little objective inflammation may provide an especially informative comparison group for distinguishing sensory-dominant from inflammation-associated patterns.

A useful initial design would compare carefully characterized patients with symptomatic controls and asymptomatic controls, with longitudinal follow-up through treatment and recovery. Sampling should identify the precise pharyngeal subsite and standardize timing, recent food or drink, oral hygiene, and sample handling. Saliva, surface swabs, epithelial brushings, and biopsies answer different biological questions. Saliva captures a broader oral environment; brushings favor surface cells; biopsies preserve tissue architecture but involve greater procedural burden. Interpreting each specimen according to its anatomical and cellular composition will be important for defining what “local immunity” represents in a given study.

Future cohorts should examine the nasopharyngeal/epipharyngeal and oropharyngeal compartments within the same participants rather than assigning the anatomical origin of inflammation from symptoms alone. Paired site-specific examination and sampling could establish how frequently inflammatory abnormalities coexist across pharyngeal regions, whether they share biological programs, and whether apparently similar symptom phenotypes arise from compartment-specific or multi-site inflammatory processes.

Age and developmental context also deserve particular attention. The human tonsil atlas demonstrates age-related compositional differences (6), and a recent rat reflux model found different IL-6 response patterns in laryngeal and laryngopharyngeal tissues (74). These observations reinforce the methodological importance of anatomical and developmental annotation. Site-resolved analysis may reveal biological heterogeneity that would otherwise be obscured by pooling adjacent tissues or age groups.

### 10.3 Connect cellular maps to functional experiments

Single-cell and spatial profiling should address prespecified questions in clinically annotated cohorts. Existing human epipharyngeal Visium HD data demonstrate the feasibility of resolving epithelial and immune-cell organization within a defined pharyngeal compartment (60). Future studies can extend this site-resolved approach across clinically defined chronic-pharyngitis phenotypes and pharyngeal subsites. Candidate cell states can then be confirmed in independent samples using imaging, flow cytometry, targeted molecular assays, or functional experiments. Spatial analyses should distinguish diffuse mucosa from organized lymphoid tissue and relate immune populations to epithelial injury, microbial localization, and repair.

Paired primary pharyngeal cultures obtained before and after clinical recovery could test whether altered epithelial responses persist outside the patient environment. Co-culture with autologous myeloid or lymphoid cells could determine which phenotypes depend on intercellular communication. Exposure intensity, recovery intervals, viability controls, and selective perturbation or rescue should be incorporated into these experiments so that cell-state maps can be linked to causal biological functions.

### 10.4 Design trials to measure recovery, not only short-term suppression

**Figure 2** outlines a proposed sequence from clinical characterization to mechanistic validation and intervention. Early trials should define a clinically meaningful symptom endpoint while incorporating local biological outcomes selected according to the mechanism under study. A barrier-directed intervention, for example, should include a validated measure of permeability or epithelial integrity. A microbial intervention should establish exposure to the administered organism or product and assess the microbial function being targeted. A sensory intervention should measure symptom and neural outcomes alongside mucosal biology.

**Figure 2:**
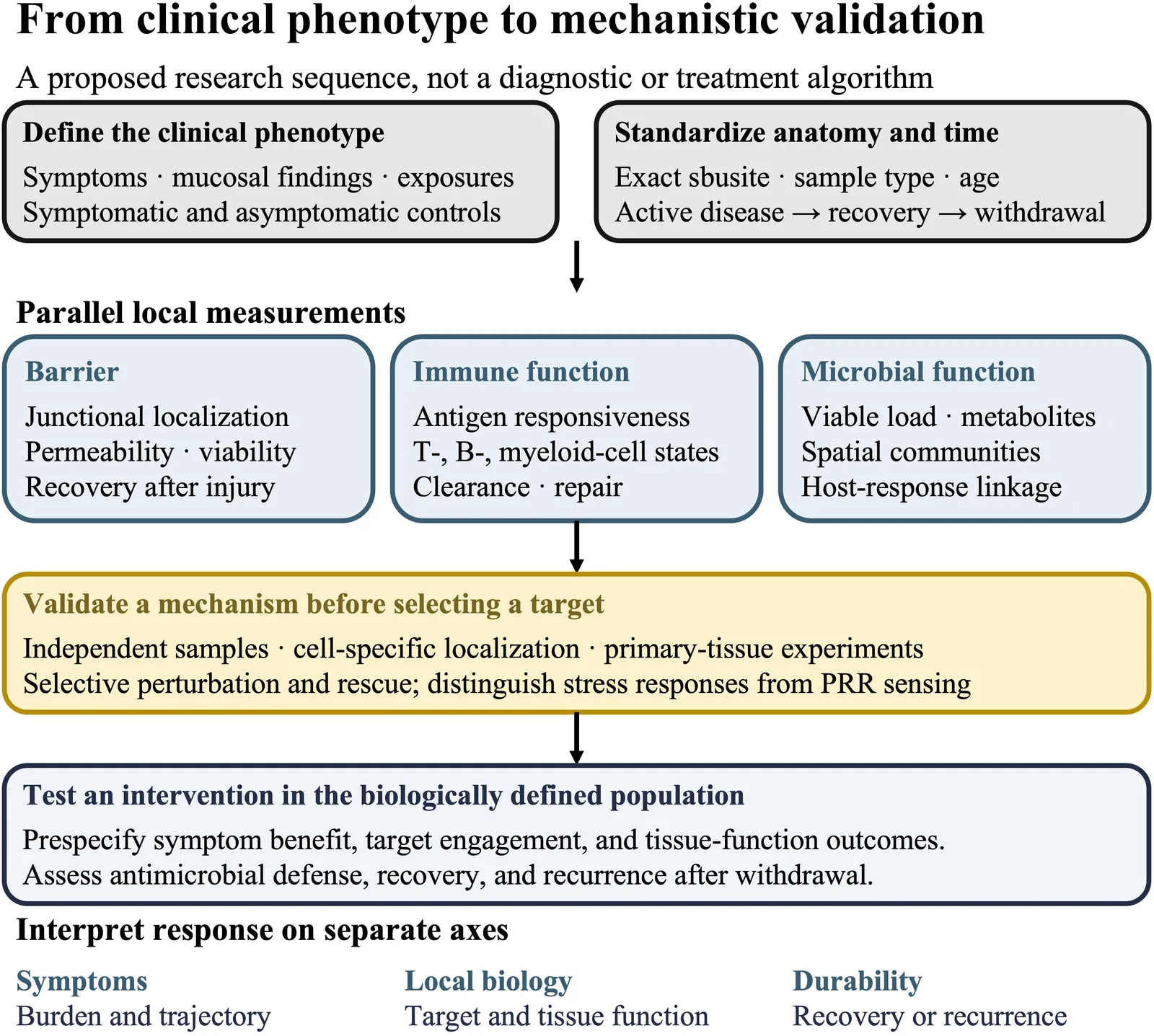
Proposed research sequence linking clinical characterization to mechanistic validation in chronic pharyngitis. Site-resolved longitudinal sampling supports parallel assessment of barrier, immune, and microbial functions. Candidate mechanisms should be validated in independent samples and primary-tissue or cell-based perturbation experiments before target selection. Interventions should be tested in biologically defined populations, with symptom benefit, local biological effects, and durability of recovery assessed as separate outcomes. Follow-up after treatment withdrawal can help distinguish transient suppression from restoration of a more stable mucosal state. Discordant symptom and biological outcomes may identify mechanisms that would be obscured by symptom-only endpoints. PRR, pattern-recognition receptor. Original conceptual schematic; no quantitative data are represented.

Follow-up after treatment withdrawal is particularly informative. Sustained symptom improvement together with restored tissue function would support durable recovery; prompt recurrence after treatment withdrawal while the initiating exposure persists may indicate temporary protection; persistent symptoms despite biological normalization may suggest a residual sensory or other symptom-generating component. Prespecifying these response patterns can convert post-treatment trajectories into mechanistically interpretable outcomes. Sample-size calculations should be based on the primary endpoint, exploratory biomarkers should be clearly designated, and molecular subgroups should be reproduced in independent cohorts before being treated as stable endotypes.

A central challenge for this field is the contrast between the breadth of plausible mucosal mechanisms and the relatively small number of deeply characterized human chronic-pharyngitis cohorts. Heterogeneous terminology, anatomical sampling sites and methods, and study designs further contribute to variability across the literature. The framework proposed here is therefore intended as a testable research architecture: its value will depend on whether it yields reproducible biological subgroups, clarifies mechanisms of persistence and recovery, and supports interventions whose clinical effects can be linked to demonstrated local processes.

## 11 Conclusion

Chronic pharyngitis is best approached as a heterogeneous clinical problem in which persistent symptoms may arise from different combinations of epithelial injury, immune activation, microbial interactions, altered tissue responsiveness, and sensory signaling. Experimental studies identify epithelial barrier disruption, innate inflammatory pathways, and impaired recovery as important mechanistic candidates, while human studies provide a foundation for defining how these processes vary across clinically characterized populations. Adaptive immunity, microbial ecology, and sensory pathways add further dimensions that can be resolved through anatomically precise and longitudinal investigation.

Progress will depend on linking clinical presentation to local biological function, assessing symptoms and mucosal inflammation as complementary outcome dimensions, and determining whether interventions restore tissue function and durable recovery rather than only modifying short-term readouts. Integrating longitudinal sampling, spatial and functional analyses, and mechanism-informed trials may ultimately support a more biologically precise classification of chronic pharyngitis and guide treatment according to the mechanisms operating in individual patient groups.
