# Supplementary material for: The mucosal immune microenvironment in chronic pharyngitis: epithelial barrier dysfunction, inflammatory persistence, and therapeutic perspectives
Source: Front Immunol. 2026 Oct 23;17:2000898. doi: 10.3389/fimmu.2026.2000898 (PMC13740825; doi:10.3389/fimmu.2026.2000898)
Supplement: Supplementary file 1 [file Table1.docx]

***Supporting Information***

**Supplementary Table S1. PubMed search strategy and retrieval counts for the revised narrative review**

All searches were conducted in PubMed on October 7, 2026, from database inception, with no lower publication-date restriction. Unless otherwise specified, terms were searched in the Title/Abstract field. Retrieval counts reflect the number of records returned by each query and should not be summed because searches overlap.

Core disease block (D): ("chronic pharyngitis"[Title/Abstract] OR "chronic hypertrophic pharyngitis"[Title/Abstract] OR "chronic atrophic pharyngitis"[Title/Abstract] OR "chronic granular pharyngitis"[Title/Abstract] OR "chronic catarrhal pharyngitis"[Title/Abstract])

| Search Domain | PubMed Search Strategy | Records Retrieved | Purpose |
| --- | --- | --- | --- |
| Core chronic-pharyngitis literature | D | 224 | Identify disease-specific literature using major chronic-pharyngitis terminology. |
| Broad sensitivity search | chronic[Title/Abstract] AND pharyngitis[Title/Abstract] | 768 | Check alternative wording not captured by the phrase-based core strategy. |
| Immune/inflammatory responses | D AND (immun*[Title/Abstract] OR inflamm*[Title/Abstract]) | 63 | Identify directly disease-linked immune or inflammatory studies. |
| Cytokines | D AND cytokine*[Title/Abstract] | 7 | Identify cytokine-related studies. |
| Lymphoid cells | D AND ("T cell"[Title/Abstract] OR "T cells"[Title/Abstract] OR "B cell"[Title/Abstract] OR "B cells"[Title/Abstract] OR lymphocyte*[Title/Abstract]) | 7 | Identify T-cell, B-cell, or lymphocyte-related studies. |
| Myeloid cells | D AND (macrophage[Title/Abstract] OR macrophages[Title/Abstract] OR neutrophil[Title/Abstract] OR neutrophils[Title/Abstract]) | 5 | Identify macrophage- or neutrophil-related studies. |
| Epithelial biology | D AND (epithelial[Title/Abstract] OR epithelium[Title/Abstract]) | 2 | Identify epithelial studies directly linked to chronic pharyngitis. |
| Barrier biology | D AND (barrier[Title/Abstract] OR permeability[Title/Abstract] OR "E-cadherin"[Title/Abstract] OR junction[Title/Abstract] OR junctional[Title/Abstract]) | 2 | Identify barrier- and junction-related studies. |
| Microbiota/microbial terminology | D AND (microbiome[Title/Abstract] OR microbiota[Title/Abstract] OR microbial[Title/Abstract]) | 4 | Identify microbiome and microbial-ecology studies. |
| Infection | D AND (infection[Title/Abstract] OR bacterial[Title/Abstract] OR bacteria[Title/Abstract] OR fungal[Title/Abstract] OR fungi[Title/Abstract]) | 31 | Identify pathogen- and infection-related literature. |
| Treatment | D AND (treatment[Title/Abstract] OR therapy[Title/Abstract] OR therapeutic[Title/Abstract]) | 108 | Characterize the broader therapeutic evidence base. |
| Randomized studies | D AND (randomized[Title/Abstract] OR randomised[Title/Abstract] OR randomized controlled trial[Publication Type]) | 16 | Identify randomized clinical evidence. |
| Systematic reviews/meta-analyses | D AND ("systematic review"[Title/Abstract] OR meta-analysis[Title/Abstract] OR meta-analysis[Publication Type]) | 6 | Identify higher-level evidence syntheses. |
